# Obese visceral fat tissue inflammation: from protective to detrimental?

**DOI:** 10.1186/s12916-022-02672-y

**Published:** 2022-12-27

**Authors:** Hubert Kolb

**Affiliations:** 1 Faculty of Medicine, University of Düsseldorf, Moorenstr. 5, 40225 Düsseldorf, Germany; 2 West-German Centre of Diabetes and Health, Düsseldorf Catholic Hospital Group, Hohensandweg 37, 40591 Düsseldorf, Germany

**Keywords:** Obesity, Adiposity, Visceral fat, Inflammation, Adipocyte hypertrophy, Adipocyte hyperplasia, Adipose tissue macrophages, Resident immune cells, Cytokines, Crown-like structures

## Abstract

Obesity usually is accompanied by inflammation of fat tissue, with a prominent role of visceral fat. Chronic inflammation in obese fat tissue is of a lower grade than acute immune activation for clearing the tissue from an infectious agent. It is the loss of adipocyte metabolic homeostasis that causes activation of resident immune cells for supporting tissue functions and regaining homeostasis. Initially, the excess influx of lipids and glucose in the context of overnutrition is met by adipocyte growth and proliferation. Eventual lipid overload of hypertrophic adipocytes leads to endoplasmic reticulum stress and the secretion of a variety of signals causing increased sympathetic tone, lipolysis by adipocytes, lipid uptake by macrophages, matrix remodeling, angiogenesis, and immune cell activation. Pro-inflammatory signaling of adipocytes causes the resident immune system to release increased amounts of pro-inflammatory and other mediators resulting in enhanced tissue-protective responses. With chronic overnutrition, these protective actions are insufficient, and death of adipocytes as well as senescence of several tissue cell types is seen. This structural damage causes the expression or release of immunostimulatory cell components resulting in influx and activation of monocytes and many other immune cell types, with a contribution of stromal cells. Matrix remodeling and angiogenesis is further intensified as well as possibly detrimental fibrosis. The accumulation of senescent cells also may be detrimental via eventual spread of senescence state from affected to neighboring cells by the release of microRNA-containing vesicles. Obese visceral fat inflammation can be viewed as an initially protective response in order to cope with excess ambient nutrients and restore tissue homeostasis but may contribute to tissue damage at a later stage.

## Background

Fat tissue is interspersed with resident immune cells as are all other solid tissues of the body. Activation of such immune cells usually is accompanied by local or systemic inflammation of varying intensity. A key characteristic of inflammation is the increased production of cytokines, chemokines, and other immune mediators which bind to cognate receptors present on/in numerous cell types in the local tissue and throughout the body. In addition to the role of resident immune cells, most all other cell types of a tissue can be activated to release pro-inflammatory mediators, usually at lower levels that seen for “professional” immune cells. Mild responses include local insulin resistance, oxidative stress and altered cell metabolism. Higher degrees of inflammation are characterized by the activation and infiltration of circulating immune cells which may cause local pain, edema, or fever [1, 2].

Triggers of resident immune cell activation not only comprise microbial or other infections but also “sterile” stimuli such as metabolic, physical, or toxic stress leading to excess production of oxygen radicals, reactive lipids, protein aggregates, stress proteins or cell necrosis followed by the release of diverse immunostimulatory compounds termed damage-associated molecular patterns [1].

The present review describes changes in visceral fat tissue in response to chronic overnutrition, the signals and cell types involved in the early stages of tissue inflammation, and the progression to full-blown inflammation characterized by tissue damage and infiltration of circulating immune cells.

## Main text

### Resident immune cells in lean visceral fat tissue

**Fig. 1:**
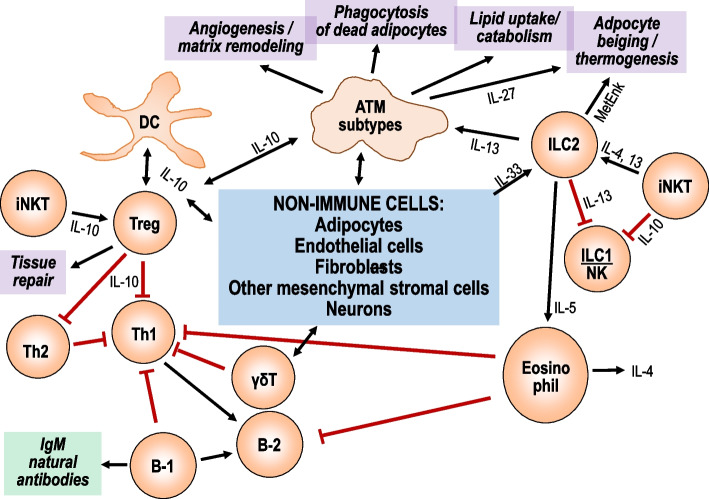
Network and physiological functions of resident immune cells in lean visceral adipose tissue. In the absence of metabolic or inflammatory stress resident immune cells interact among themselves and with adipocytes and stromal cells to maintain proper tissue functions. There are no signature cytokines defining the maintenance state of resident immune cells. Rather, the concept of a buffered system applies, without polarization towards a Th1/M1- or Th2/M2-like pattern or towards another biased state of immune reactivity. Cytokines, chemokines, acute phase proteins, and other immune mediators are released in small amounts mostly from resident immune cells but also from mesenchymal stromal cells and adipocytes. Several macrophage subtypes promote matrix remodeling and angiogenesis, phagocytose dead cell and lipid aggregates, and promote adipocyte thermogenesis. ILC2 also supports adipocyte thermogenesis and stimulates physiological eosinophil functions. Regulatory T cells promote tissue repair and interact with macrophages and other immune cell types to maintain a non-inflammatory state. Low-level secretion of immune mediators by macrophages, dendritic cells, and other immune cell types such as ILC2s, iNKTs, Th2 cells, γδT cells, B-1b cells, and eosinophils helps to prevent immune cell activation. For better readability, only a few key intercellular signals are included in the scheme. ATM, adipose tissue macrophage; DC, dendritic cell; IL, interleukin; ILC, innate lymphoid cell; iNKT, innate natural killer T cell; MetEnk, methionine-enkephalin peptides; NK, natural killer cell

In visceral fat, members of the innate as well as adaptive immune system have been identified. These include macrophages, dendritic cells, granulocytes, innate lymphoid cells (ILCs and natural killer (NK) cells), and also T and B cells [3] (Fig. 1). Single-cell transcriptome analysis of mouse lean visceral adipose tissue leukocytes identified 15 distinct subpopulations [4]. In normal non-inflamed tissue, these cells are not only immune guardians against infection but also support proper tissue function. Many of these findings originate from studies in experimental models, but where analyzed, similar physiological functions of immune cells have also been reported in humans [5]. For instance, macrophages exhibit functional heterogeneity which includes the removal of dead or apoptotic fat cells, remodeling the extracellular matrix and promoting angiogenesis [5]. A subtype of macrophages supports the control of lipid metabolism by uptake and digestion of lipids [6, 7]. Furthermore, the secretion of IL-27 appears to be a major pathway of promoting thermogenesis in fat cells [8]. Macrophages contribute to the regulation of thermogenesis in response to cold exposure [9]. There is no homogeneous distribution of macrophage subtypes. For instance, in human subcutaneous tissue, spatial mapping identified macrophages with a M1-like phenotype associated with niches of adipocyte progenitor cells while macrophages with a non-inflammatory phenotype were dispersed throughout the fat tissue [10].

ILC2 cells contribute to the regulation of energy expenditure by promoting the differentiation of beige adipocytes from adipocyte precursors or beiging of white fat cells in visceral tissues via upregulation of uncoupling protein 1 (UCP1 by enkephalin peptides [11, 12]. ILC2-derived IL-13 helps to prevent pro-inflammatory activation of macrophages, dendritic cells, ILC1, and natural killer cells. By secreting IL-5, ILC2 cells promote anti-inflammatory eosinophil activity.

Conventional dendritic cells exhibit a tolerogenic phenotype, characterized by IL-10 production and suppression of Th1-promoting activity by upregulated expression of peroxisome proliferator-activated receptor gamma (PPAR-γ) [13]. Regulatory T cells (Treg) represent the major CD4-positive T cell type, they participate in tissue repair and preserve Glut-4 expression by adipocytes [14, 15]. Interestingly, the majority of Tregs appear to be oligoclonal in mice as indicated by distinct T cell receptor repertoires. There may be MHC II-dependent antigen recognition involved, as suggested by the close association with resident macrophages and dendritic cells [16]. The primary function of Tregs probably is to keep other immune cell types in a neutral physiological state, i.e., preventing immune activation either towards a pro-inflammatory or a Th2-like state.

Resident B-1b lymphocytes secrete natural IgM antibodies and promote adipose physiological functions by suppressing B-2 cells, in mice and humans [17]. In addition, B-1 cells comprise the major cell type of fat-associated lymphoid clusters which appear to contribute to humoral immune responses to peritoneal antigens [18]. Lymphoid clusters in mice and humans are also a rich source of Th2-like cytokines released from innate Th2-like lymphoid cells [19, 20]. Fat-associated lymphoid clusters such as milky spots on the omentum surface probably serve immune functions of the peritoneal cavity rather than supporting physiological fat tissue functions. Indeed, the numbers of milky spots increase during peritoneal inflammation in response to local TNFα and innate natural killer T cell activity [20, 21]. Studies in mice suggest that sympathetic innervation is promoted by γδT cells by signaling via the IL-17 receptor C to induce TGFß1 production by parenchymal cells [22]. Further, sympathetic neuron-associated macrophages (SAMs) regulate neuron growth and modulate adrenergic signaling [23].

Based mostly on animal studies, the continuous release of anti-inflammatory mediators from macrophages, dendritic cells, Th2-cells, γδT cells, eosinophils, mucosa-associated invariant T cells (MAIT), and invariant natural killer T cells appears to further help maintain metabolic homeostasis [14, 24–32] (Fig. 1). The support of tissue functions by resident immune cells involves interactions with non-immune tissue cells including adipocytes, endothelial cells, neurons, fibroblasts, and other mesenchymal stromal cells [3, 33, 34].

In the absence of immunologic stimuli, immune mediator secretion from resident immune cells and other fat tissue cells is low. The local immune milieu is well buffered, i.e., there is neither a dominance of Th1/M1-like nor Th2/M2-like immune reactivity. In this context, it is important to note that there is interdependence of Th1/M1- and Th2/M2-associated cytokines. For instance, the Th1-type cytokine TNFα stimulates the production of the Th2/Treg-associated cytokine IL-10 which in turn downregulates TNFα. Further, pro-inflammatory TNFα and IL-17A induce counterregulatory IL-33 for the stimulation of anti-inflammatory Tregs and ILC2s [15, 35, 36].

Taken together, in lean visceral adipose tissue, there is a physiological network of adipocytes, stromal cells, and immune cells. The resident immune system is not dormant but supports overall tissue functions. There are no signature cytokines defining the maintenance state of resident immune cells. Rather, the concept of a buffered system applies, without polarization towards a Th1/M1- or Th2/M2-like pattern or towards another biased state of immune reactivity. Cytokines, chemokines, acute phase proteins, and other immune mediators are released in small amounts mostly from resident immune cells but also from mesenchymal stromal cells and adipocytes [37, 38].

### From lean to obese visceral fat tissue

The primary cause of progression from lean to obese visceral fat tissue is excess calorie intake, including digestible carbohydrates. Human metabolic control usually is geared in such a way that a calorie surplus is not disposed of by generating additional thermal energy but is stored to a large degree as triglycerides in adipocytes. Excess calorie consumption causes an increase of circulating insulin levels after and between meals. Being an anabolic hormone, insulin suppresses lipolysis and promotes fat storage in adipocytes already at concentrations that are in the high normal range or which are slightly elevated. Pharmacological or experimental lowering of insulin levels indeed ameliorates obesity which indicates that the support of lipogenesis by insulin is obesogenic (reviewed by [39]). These regulatory effects of insulin do not apply for all adipocytes. In subcutaneous tissue about half of mature adipocytes are insulin responsive, the two other subtypes exhibit little or no increased transcriptional activity when exposed to hyperinsulinemia [10].

**Fig. 2:**
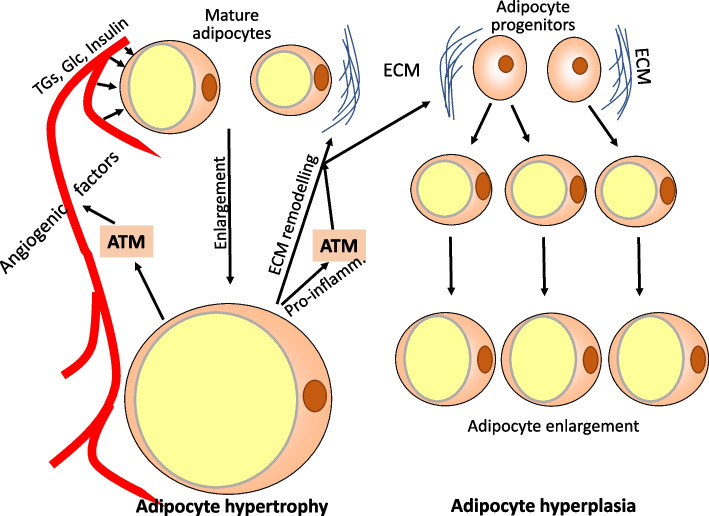
Response of visceral fat tissue to excess calories by adipocyte hypertrophy and hyperplasia. In response to high levels of circulating glucose, triglycerides, and the anabolic hormone insulin mature adipocytes take up increased amounts of nutrients and store excess energy as triglycerides in one large lipid droplet organelle. The cell size may increase 10–15-fold in diameter. Enlarged adipocytes secrete factors favoring angiogenesis and remodeling of the extracellular matrix and release of growth factors which is essential for mesenchymal stem cells, adipocyte progenitors, and preadipocytes to differentiate into lipid-storing mature adipocytes. In parallel, macrophages are stimulated to support angiogenesis and matrix remodeling. ATM, adipocyte tissue macrophages; TGs, triglycerides; Glc, glucose; ECM, extracellular matrix; Pro-inflamm., pro-inflammatory mediators

Anabolic activity of visceral fat tissue in response to overnutrition involves adipocyte enlargement and hyperplasia to accommodate for increased requirements of energy storage, i.e., clearance of excess lipids and glucose from the blood. Lipogenesis leads to enlargement of mature adipocytes because of more fat stored in one large lipid droplet organelle. The gain in cell volume may be several thousandfolds, with a cell diameter increase from <20 up to 300 μm [40]. There is also differentiation and growth of preadipocytes, but in visceral fat hyperplasia contributes less to the increase of fat mass than adipocyte hypertrophy [41]. The formation of new fat-laden adipocytes from precursor cells appears to begin when enlarged mature adipocytes reach a critical cell size and release mediators stimulating preadipocyte growth and differentiation [42, 43]. Fat cell hyperplasia thus is a second pathway of coping with excess circulating nutrients (Fig. 2) [43].

Studies in mice indicate that a major obstacle to fat tissue expansion in response to high-fat diet feeding is the collagen network of the extracellular matrix. A major source of collagen is perivascular cells in response to signaling via the platelet-derived growth factor 1α [44]. Most relevant for limiting fat tissue expansion is the extracellular matrix of niches rich in adipocyte precursors. Interestingly, these niches harbor potentially pro-inflammatory macrophages [10] and induction of acute local inflammation, for instance by injection of low-dose lipopolysaccharide enhances fibrolysis and remodeling of the extracellular matrix, and promotes angiogenesis to allow for efficient adipocyte hyperplasia. Enlarged adipocytes initiate fat tissue remodeling by secreting angiogenic factors such as such as fibroblast growth factor-2, vascular endothelial growth factor, human growth factor, and other mediators such as extracellular matrix proteases (Fig.2) [41]. Efficient remodeling requires activation of pro-inflammatory macrophages by hypertrophic adipocytes which appears to be a physiological response needed for fat tissue growth because downregulation of pro-inflammatory reactivity prevents proper adipocyte hyperplasia [41, 45, 46]. Thus, at least initially, inflammation in adipose tissue is a physiological adaptive response which improves fat tissue plasticity and consequently preserves metabolic control and insulin sensitivity [47]. A similar important role of inflammatory reactions, such as activation of the NLRP3 inflammasome, has been reported to drive postburn white adipose tissue remodeling [48].

Storage of energy in form of triglycerides also occurs in other fat tissues of the body, notably subcutaneous fat. The adipogenic activity and the ability to mobilize preadipocytes in response to overeating have been reported to be delayed in subcutaneous fat and therefore may be insufficient to lower the metabolic stress of visceral fat tissue during excess calorie intake [43]. However, this is different in persons with true metabolic healthy obesity, i.e., defined by an absence of metabolic syndrome criteria (except for increased waist circumference) and of insulin resistance calculated as HOMA-IR [49]. In these persons, the growth of visceral fat and adipocyte enlargement is only moderate, and excess nutrients are primarily handled by enlargement and hyperplasia of adipocytes in subcutaneous fat tissue, primarily in the superficial layer [43, 50–52].

In sum, the primary fat tissue response to excess calorie intake includes enlargement of adipocytes, differentiation of new mature cells from pre-adipocytes or stem cells, all supported by remodeling of the extracellular matrix, and of angiogenesis for appropriate blood supply. Growth of visceral fat tissue is not possible without appropriate remodeling of the vasculature and the extracellular matrix surrounding preadipocytes and small adipocytes. Enlarged adipocytes initiate these changes by secreting factors promoting angiogenesis and matrix remodeling. These adaptive responses are characteristic of metabolically healthy obesity.

### Obese visceral fat tissue inflammation in response to disturbed local metabolic homeostasis

A recent overview of inflammatory responses to non-infectious stimuli in various tissues of the body has concluded that there appear to be three types of perturbation causing an inflammatory response which, at least initially, are considered protective [2] The suggested hierarchy of perturbations is loss of regulation, loss of function and loss of structure. This concept is applied here to obese fat tissue, and the current section considers loss of regulation.

In those visceral adipose regions where the adaptive response to excess energy influx has reached a limit, metabolic homeostasis is lost, and activation of resident immune cells occurs. In detail, strongly enlarged adipocytes fail to maintain metabolic homeostasis of lipid storage versus lipolysis because the lipid overload leads to endoplasmic reticulum stress, increased expression of the inflammation regulator NF-kB and the production of inflammation-inducing signals such as IL-6 [40, 53]. The secretion of pro-inflammatory mediators in response to loss of metabolic homeostasis has been termed metaflammation [54].

Enlarged adipocytes exhibit additional responses to caloric stress. For instance, adipocytes respond to high ambient nutrient concentrations with the release of leptin and other hormones which target the brain to limit food intake and increase the sympathetic tone. Adrenaline and noradrenaline are released from nerve endings in adipose tissue and activate lipolysis by signaling via ß-adrenergic receptors of adipocytes. Sympathetic neuron-associated macrophages may function as rate-limiters by degrading noradrenaline via monoamine oxidase A [23]. The locally increased concentration of non-esterified fatty acids is expected to activate pro-inflammatory macrophage functions. This may involve co-secretion of adipocyte fatty acid binding protein (FABP4), induction of FABP4 in macrophages, and signaling via toll-like receptors TLR4 and TLR2. Free fatty acids do not directly bind to TLR4, but lipid metabolism within macrophages is affected by the influx of free fatty acids which has pro-inflammatory consequences if there is simultaneous activation of TLR4. The latter may result from increased levels of lipopolysaccharide released from gut microbiota in the context of gut leakiness during an obesogenic diet [55–60]. Further, recent studies suggest a role of adenine nucleotide translocase 2 in mediating free fatty acid-induced mitochondrial dysfunction, increased oxygen radical production and NF-kB activation in fat tissue macrophages [61]. The secretion of leptin by enlarged adipocytes not only limits food intake and promotes lipolysis in visceral fat but also engages leptin receptors present on most immune cells. This results in modest activation of immune reactivity of resident immune cells towards a Th1/M1-type pro-inflammatory bias [54, 62].

**Fig. 3:**
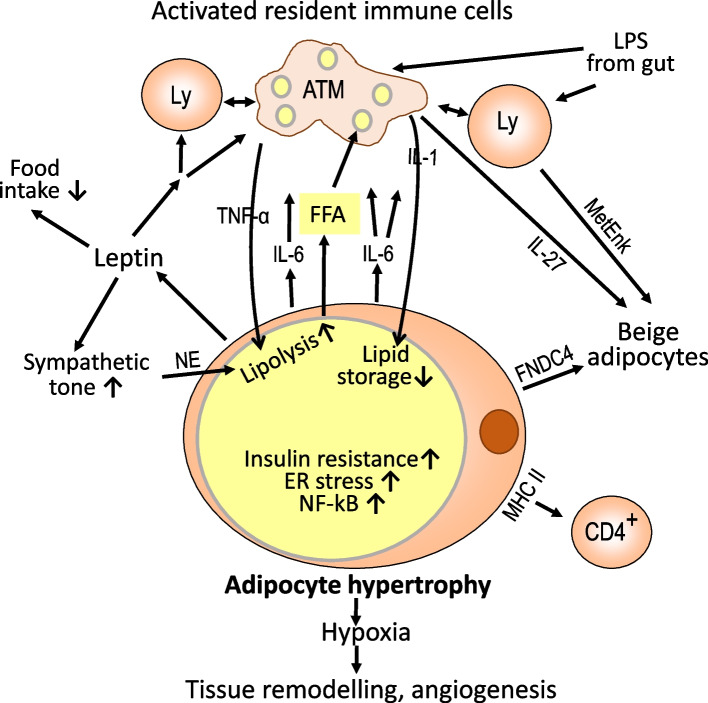
Local inflammation in response to disturbed adipocyte metabolic homeostasis. When enhanced lipid storage via adipocyte enlargement and differentiation of progenitor cells fails to maintain metabolic homeostasis, local inflammatory changes occur in order to dispose of excess lipid and regain metabolic control. For one, lipid-laden adipocytes experience endoplasmic reticulum stress and increased expression of NFkB leading to the release of pro-inflammatory mediators such as IL-6. Additional pro-inflammatory signals are delivered by the release of free fatty acids, leptin, lipopolysaccharides, and other products of an unbalanced microbiota in the context of a leaky gut. Activated resident immune cells release amounts of pro-inflammatory mediators sufficient to promote lipolysis and suppress lipid storage in part via induction of insulin resistance. In addition, there is an uptake of lipids by macrophages and storage in small lipid droplets. Leptin interacts with receptors in the brain to limit food intake and increase the sympathetic tone. The increased local release of noradrenaline also promotes lipolysis. Another pro-inflammatory condition results from hypoxia due to local enlargement of adipocytes. The concomitant release of enzymes and factors promoting tissue remodeling and angiogenesis may be considered a healing response. Enlarged adipocytes overexpress MHC class II antigens and appear to present antigens to CD4-positive T cells. Another pathway of limiting energy storage is the induction of adipocyte beiging by transdifferentiation or growth from progenitors and the disposal of excess energy by thermogenesis. For better readability, only a few key intercellular signals are included in the scheme. FNDC4, fibronectin type III domain-containing protein 4; FFA, free fatty acids; LPS, lipopolysaccharide; NE, norepinephrine/noradrenaline; NFkB, nuclear factor kappa B

Another pathway of promoting local inflammation in response to adipocyte enlargement is activated by rapid fat tissue growth in the presence of insufficient angiogenesis which lowers capillary density and increases diffusion distance for oxygen eventually resulting in a hypoxic environment of enlarged adipocytes. Adipose is among the most vascularized tissues with each adipocyte surrounded by capillaries [63]. Lowering ambient oxygen concentration in adipocyte culture caused a switch from oxidative phosphorylation to anaerobic glycolysis and changed the expression of more than 1000 genes [64]. One major mediator of this response is hypoxia-inducible factor 1α [55]. Pro-inflammatory mediators secreted by mature adipocytes during hypoxia include chemokines and cytokines such as PAI-1, CCL5, and IL-6 as well as micro RNAs [65–68] (Fig. 3). A subset of macrophages is closely associated with the vasculature and characterized by the expression of lymphatic vessel endothelial hyaluronan receptor 1. These macrophages support angiogenesis by producing tissue remodeling growth factors and metalloproteinases [21, 69]. Hypoxia does not homogeneously affect visceral fat tissue but is a regional phenomenon as concluded from immunohistochemical staining for hypoxia-inducible factor 1α. The colocalization of enhanced numbers of macrophages and T cells supports the pro-inflammatory property of hypoxia [70].

A third pathway of pro-inflammatory activation of resident immune cells is suggested by the finding that hypertrophic adipocytes overexpress major histocompatibility antigens class II (MHCII) and produce costimulatory molecules for effective antigen presentation to CD4 positive T cells. Although antigens presented have not been identified, it is remarkable that mice with genetic depletion of MHC II in adipocytes gain weight as control mice but do not develop adipose tissue inflammation and insulin resistance [71].

An additional pathway of lowering the metabolic stress in obese visceral fat tissue is the transdifferentiation of white adipocytes to beige adipocytes and the formation of new beige adipocytes from precursor cells (Fig. 3). Beige adipocytes contain several smaller lipid droplet organelles and more mitochondria than hypertrophic adipocytes for burning free fatty acids to generate heat. Secretion of IL-27 from macrophages promotes thermogenesis in fat cells [8], as does the release of enkephalin peptides from ILC2 cells [11, 12]. The major mediator of beiging in visceral fat released by adipocytes in obesity is fibronectin type III domain-containing protein 4 (FNDC4) which probably targets the receptor GRP116. There is a positive association between the expression of FNDC4 and obesity-associated inflammation [72]. In line with a role in regaining normal tissue homeostasis, FNDC4 exhibits anti-inflammatory properties in macrophages [73].

Taken together, loss of metabolic homeostasis in fat tissue is sufficient to initiate a local pro-inflammatory response. Secretion of pro-inflammatory mediators from macrophages and other immune cells substantially exceeds the release from adipocytes [38]. This may be viewed as an attempt to restore proper energy balance [74]. The locally enhanced concentrations of mediators like TNFα, IL-1, and IL-6 act back on adipocytes and suppress further lipid storage by inhibiting lipoprotein lipase, needed for lipid uptake, and by promoting lipolysis and fatty acid release via several pathways. These include the local induction of insulin resistance in insulin-sensitive adipocytes resulting from engaging TNFα or other pro-inflammatory mediators including microRNAs and subsequent impairment of insulin signaling for lipolysis inhibition [26, 75]. Further support comes from increased activation of extracellular signal-regulated kinase (ERK) stimulating beta3 adrenergic receptor-mediated lipolysis via protein kinase A [76]. Inflammatory stress induces kinase activity of inositol-requiring protein 1 (IRE-1), a component of the endoplasmic reticulum stress response, which is also followed by enhanced lipolysis [77]. In addition, there is upregulation of lysosomal biogenesis, increased uptake and turnover of lipids, and increased formation of lipid droplets in macrophages, all of which can be considered an attempt to lower the lipid load of adipocytes [78]. Finally, burning of free fatty acids via promoting thermogenesis/beiging is supported by the release of IL-27 from macrophages, enkephalin peptides from ILC2s, and FNDC4 from adipocytes. The interaction between the various cell types in adipose tissue can also be described as crosstalk since there is signaling between cells in both directions. Crosstalk not only involves the secretion of soluble mediators but also of particulate structures such as extracellular vesicles or mitochondria [79, 80].

The scenario described relates to observations in animal models. In humans, the direct demonstration an early phase of inflammatory reactions induced by metabolically stressed enlarged adipocytes during overnutrition would require repeated biopsies of visceral fat tissue, but the mechanisms detailed above also apply to human cells. In mice, high-fat diet feeding studies observed an early period of 4–8 weeks with adipocyte enlargement, limited local immune activation, vasculogenesis, matrix remodeling, and clearance of a low number of dead adipocytes by local macrophages [81].

### Influx of immune cells into obese visceral fat inflammation in response to functional/structural tissue damage

A general characteristic of tissue damage is the loss of structural integrity, i.e., molecular cues are presented on cell surfaces or are released that are usually sequestered and not accessible to the immune system. Many of these molecules are immunostimulatory damage-associated molecular patterns (DAMPs), they include stress proteins, high mobility group box 1 protein (HMGB1), DNA, some lipids, and mitochondrial structures, among many others. DAMP receptors (also called pattern recognition receptors) are present on innate immune cells and in part also on adaptive immune cells and non-immune cells such as epithelial cells, endothelial cells, or fibroblasts. DAMP receptors include toll-like receptors, C-type lectin receptors, cytoplasmic NLR receptors, and several DNA sensors. Signaling via these receptors leads to the production of pro-inflammatory cytokines and other mediators [82, 83].

In visceral fat tissue, major sources of DAMPs are apoptotic/necroptotic/pyroptotic adipocytes. Dead adipocytes accumulate in obese visceral tissue and attract resident macrophages giving the image of crown-like structures resulting in phagocytic activity and proliferation. Apoptotic adipocytes express surface proteins favoring phagocytosis by M2-type macrophages [84]. Treg cells also associate with crown-like structures and probably support non-inflammatory macrophage functions [14]. However, probably because of the size difference of macrophages and hypertrophic dying adipocytes, there is also lysosomal exocytosis, and the released DAMPs stimulate pro-inflammatory immune activities which are more M1- than M2-like [85]. Immune activation by DAMPs appears to exceed pro-inflammatory signaling caused by metabolically stressed adipocytes because there is an influx of monocytes and other immune cells which outnumber resident immunocytes [55, 84]. In mouse fat tissue, induction of inflammasome and caspase-1 activity for the release of IL-1 and IL-18 is required for the recruitment of circulating immune cells and their pro-inflammatory activation [86]. Secretion of macrophage chemotactic protein 1 (MCP-1) also contributes to monocyte attraction [87, 88].

Another functional/structural change in obese visceral tissue is the accumulation of senescent cells, mostly macrophages, pre-adipocytes, mature adipocytes, and endothelial cells, probably in response to high metabolic activity, concomitant high oxygen radical production, mitochondrial dysfunction, and DNA damage. These cells exhibit impaired cell functions and an irreversible proliferative arrest in association with the secretion of a variety of pro-inflammatory cytokines, chemokines, proteases, and vesicles containing microRNAs, DNA, lipids, and protein. Peptides secreted in the context of the senescence-associated secretory phenotype (SASP) not only stimulate adipocytes and activate resident immune cells but also help recruit circulating immune cells to fat tissue followed by their activation [58, 89–91].

Structural damage also ensues if physiological remodeling of the extracellular matrix of obese visceral fat tissue is insufficient to adapt to tissue growth and enhanced angiogenesis. Collagen accumulates around adipocytes and in fiber bundles leading to decreased tissue plasticity. This leads to an adipocyte-mediated release of endotrophin, a cleavage product of collagen VI, which enhances local inflammatory responses [92–94].

**Fig. 4:**
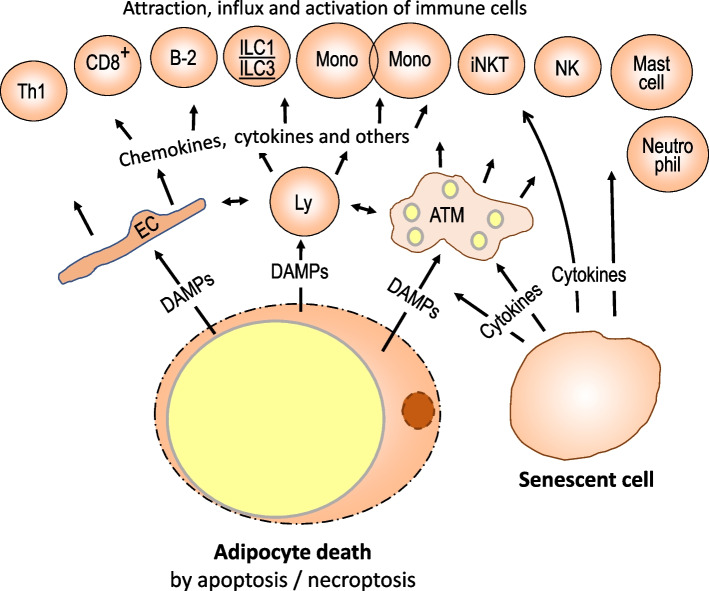
Severe visceral fat tissue inflammation in response to structural disruption. Excessive enlargement of adipocytes in response to chronic overnutrition eventually causes structural damage with dying adipocytes and cell senescence as hallmarks. The phagocytotic capacity of macrophages is overwhelmed and released DAMPS strongly activate resident immune and endothelial cells resulting in the attraction of virtually all types of immune cells. Their pro-inflammatory activation also stimulates anti-inflammatory activities. Another structural change is the accumulation of senescent cells, mostly macrophages, pre-adipocytes, mature adipocytes, and endothelial cells. Senescent cells secrete pro-inflammatory mediators and enhance the accumulation of immune cells from circulation. ATM, adipose tissue macrophages; DAMP, damage-associated molecular pattern; Mono, monocytes; EC, endothelial cell; CD8^+^, CD8-positive T cells

The findings described above suggest that the recruitment of immune cells and their accumulation occurs in response to structural damage of visceral fat tissue (Fig. 4). The dominant immune cells in the infiltrate are monocytes developing into tissue macrophages. Concomitantly, there is an influx of other immune cell types, including T and B cells, ILC1s, ILC3s, NK cells, mast cells, and neutrophils [25, 30, 94–99]. Since the fat tissue is not homogeneous with regard to vascularization, hypoxia, and adipocyte death, there is regional diversity of the inflammatory state.

DAMPs and free fatty acids do not exhibit the same strong immunostimulatory activity as seen for bacterial or viral components. Therefore, the inflammation quality is characterized by lesser pro-inflammatory signaling which is related but not identical to classical Th1/M1-type activity, and there is an upregulated lipid metabolism [6, 55, 100, 101]. Th1/M1-like inflammatory activity is also promoted by local strong hypoxic conditions in the context of anaerobic glycolytic immunometabolism [102, 103].

In inflamed obese visceral tissue infiltrated by immune cells, there is an overall dominance of pro-inflammatory activity. This favors pro-inflammatory versus tissue-supporting or anti-inflammatory macrophages, Th1 versus Th2 promoting dendritic cells, Th1 versus Treg cells, B-2 versus B-1b lymphocytes, ILC1s versus ILC2s and iNKT cells, mast cells and neutrophils versus eosinophils, and CD8^+^ - versus γδT cells [5, 25, 28, 29, 70, 104] (Fig. 4). However, it must be considered that any Th1/M1-type pro-inflammatory activity gives rise to an antagonistic anti-inflammatory response so that both, pro- and anti-inflammatory activities, including Th1- and Th2-associated cytokines, are upregulated. This situation is best researched for macrophages, which remain the prominent immune cell type in inflamed obese visceral tissue with structural damage, largely due to the recruitment of monocytes from circulation. Most infiltrated macrophages are polarized towards a pro-inflammatory phenotype which only partially resembles classic M1-like activity characterized by the secretion of IL-1ß, IL-18, TNFα, chemokines, and proteases [100]. As discussed above, pro-inflammatory cytokines such as TNFα elicit the production of anti-inflammatory cytokines such as IL-10 or of prostaglandin E2. A fraction of macrophages exhibits a M2-like or a mixed M1/M2 phenotype [85, 105, 106]. There is regional diversity between macrophages within and outside crown-like structures, and in other human obese visceral adipose tissue [85]. For instance, macrophages with adipogenic and angiogenic gene expression patterns are distributed more evenly in the visceral fat tissue while lipid-laden pro-inflammatory macrophages are associated with dead adipocytes [85].

Obesity induced by long-term feeding of a high-fat diet in mice also changes the major phenotype of dendritic cells in visceral fat towards a pro-inflammatory profile. There is secretion of IFNα from plasmacytoid dendritic cells [58]. In parallel, the number of regulatory T cells, supporting the maintenance function of immune cells, is decreasing. The loss of Treg lymphocytes from obese visceral tissue appears to be a direct consequence of IFNα action [58]. The lower number of regulatory T cells may be the major reason accounting for a pro-inflammatory shift in several other immunocytes. Early changes include an influx of pro-inflammatory T cells and of B lymphocytes. In high-fat diet-induced obesity of mice both cell types appear to precede peak macrophage infiltration [107, 108]. IFN γ secretion by CD4- and CD8-positive T lymphocytes as well as of NK cells and ILC1s probably is a strong activator of pro-inflammatory macrophage activity. Stimulation of T-cells for IFN γ production probably is supported by the pro-inflammatory B2 subset of B lymphocytes while the percentage of anti-inflammatory B1 cells is decreased [55, 97, 104, 107–111]. There is also activation of MAIT cells which promotes macrophage activation by secretion of TNFα and IL-17 [112].

In the context of visceral obese fat tissue inflammation, there is also an increase of activated neutrophils. These cells release extracellular traps which interact with other immune cells to promote pro-inflammatory responses and possibly contribute to remodeling of the matrix because of the protease content of traps, in addition to promoting insulin resistance [113, 114]. Obese visceral fat tissue also harbors increased numbers of mast cells [115] but it is not clear whether these cells promote or dampen inflammation [116].

The immune cell influx in response to structural damage of fat tissue appears to exhibit tissue-protective and also detrimental properties. Fat tissue repair such as elimination of dying adipocytes, enhanced lipolysis, tissue remodeling, and angiogenesis represent beneficial functions of infiltrated and resident immune cells. However, animal studies indicate that matrix remodeling during chronic inflammation eventually may lead to fibrosis, i.e., excess incorporation of fibrils such as collagen vi into the extracellular scaffold of adipocytes which limits adipose plasticity and metabolic function [117]. An alternative view suggests that a rigid extracellular membrane prevents excessive enlargement of adipocytes and supports metabolic homeostasis [118].

Senescent cells in inflamed tissue probably also have beneficial and as well as detrimental effects. In animal models, beneficial effects include the orchestration of tissue remodeling through the secretion of pro-inflammatory factors. Senescent cells positively impact health span, liver, and vascular tissue fibrosis, and wound healing [119, 120]. However, if senescent cells are not cleared within days or weeks by innate immune cells, they accumulate and spread senescence to neighboring and distant cells, mostly via secretion of microRNA-containing vesicles with the consequence of a pro-fibrotic state and deficient tissue function in hypertrophic obesity mice [46, 121–123]. Obesity and hyperinsulinemia also drive the senescence of adipocytes or visceral fat macrophages in humans [91, 124]. In obese mice, genetic or pharmacological elimination of senescent cells promoted adipogenesis and decreased the influx of monocytes into abdominal fat [89, 125]. When human obese visceral tissue containing senescent cells was transplanted into immunodeficient mice, lower glucose tolerance and increased insulin resistance were observed. These detrimental effects were suppressed by clearing the human fat tissue from senescent cells by treatment with a selenolytic cocktail prior to transplantation [90].

Severe visceral obesity often is accompanied by systemic low-grade inflammation, insulin resistance, glucose intolerance, and other measures of metabolic disturbances. This does not simply appear to be a spill-over effect because there seem to be contributions of other organs such as the liver, the hypothalamus, and the gut microbiota [126–128]. Overnutrition and excess systemic nutrients cause changes in the liver related to those described for visceral fat. There is enhanced lipid uptake by several cell types followed by disturbed metabolic homeostasis as evident from endoplasmic reticulum stress in hepatocytes. Eventually, this leads to structural tissue damage such as death of hepatocytes and fibrosis. Loss of metabolic homeostasis and tissue damage is accompanied by activation of the resident immune system. Pro-inflammatory responses are carried by Kupffer cells, stellate cells, many infiltrated immune cell types, other stromal cell types, and also by hepatocytes [129–136]. In animal models, immune intervention trials often have led to improved metabolic control with or without decreased adiposity indicating a pathogenic role of inflammatory immune reactivity [137–139]. However, most studies do not allow to distinguish between effects mediated at the level of the liver, pancreas, vasculature, gut, brain, or adipose tissue. A detailed discussion of diet-induced inflammatory changes outside the visceral fat tissue and of immune intervention studies is outside the scope of this paper.

## Conclusions (Table 1)

Inflammation of tissues in the absence of infectious, toxic or allergenic agents in general is caused by the local expression of immunostimulatory molecules in the context of metabolic or physical tissue damage. The activation of resident immune cells as well as the influx of immune cells from circulation into stressed tissue can be interpreted as an attempt to regain the previous physiological balance [2, 74]. Loss of metabolic tissue homeostasis appears to be sufficient to produce a “physiological“ local inflammatory response that enforces restoration of homeostasis. Loss of structure (tissue damage) elicits a more intense form of inflammation with influx of circulating immune cells, again primarily supporting tissue functions [2].

**Table 1:** Key messages and gaps of knowledge

• In normal non-inflamed fat tissue, resident immune cells interact with adipocytes and stromal cells for metabolic homeostasis, thermogenesis, and cell turnover. • Adipocytes deal with excess ambient nutrients by increased lipid storage (hypertrophy) and proliferation (hyperplasia). Resident pro-inflammatory macrophages support matrix remodeling required for adipocyte hyperplasia. • Excessive enlargement of adipocytes causes metabolic stress and the release of pro-inflammatory mediators which activate resident immune cells for enhanced production of immune mediators, resulting in enhanced sympathetic tone, lipolysis in adipocytes, lipid uptake in macrophages, matrix remodeling, and angiogenesis. • Chronic overnutrition eventually leads to structural/functional damage, i.e., adipocyte death and accumulation of senescent cells. Both are strong immunostimulatory processes causing the influx of monocytes and many other immune cell types from circulation. There is further enhanced matrix remodeling including fibrosis, angiogenesis, lipid uptake by macrophages, clearing of the tissue from cell debris, and spread of senescent state from affected to neighboring cells by the release of microRNA-containing vesicles. • Although tissue inflammation serves the restoration of a physiological functional state it is not known at which stage and by what quality chronic inflammation may mediate detrimental effects. • The role of senescent cells in supporting or damaging tissue functions requires further research. • Subtypes of visceral obesity remain to be defined, with age, sex and genetic background as important parameters. The protective versus detrimental functions of inflammation may differ between subtypes. • Molecular mechanisms are often deduced from animal studies. Differences between animal models and obese humans must be taken into account.	

In obese visceral fat tissue, adaptive or repair functions of macrophages and other activated immune cells include support of matrix remodeling and angiogenesis by secretion of proteases and growth factors to accommodate for adipocyte enlargement and hyperplasia, lipid uptake and catabolism to lower lipid load, stimulation of thermogenesis for lipid burning, promotion of lipolysis and local insulin resistance to reduce lipid storage, and clearance from dead adipocytes and senescent cells. Concomitant fibrosis may be regarded as protective or detrimental, and a low density of senescent cells may favor matrix remodeling. The increase of crown-like structures and the accumulation of senescent cells suggest that repair functions become overwhelmed. Whether pro-inflammatory activities carried by the immune cell infiltrate from circulation eventually contribute to tissue damage remains to be analyzed.

Finally, subtypes of visceral obesity remain to be defined, and not all of them may be represented by animal models. Subtypes may differ with respect to metabolic characteristics, age, sex or genetic background. The protective versus detrimental functions of inflammation may differ between subtypes.

## Data Availability

Data for this review were identified by searches of MEDLINE, PubMed, and references from relevant articles using the search terms “visceral fat tissue,” “fat inflammation,” “obesity inflammation,” “resident immune cells,” and “adipose tissue macrophages.” In order to limit the number of references, more recently published papers referring to several previously published articles were cited, if possible. Only articles published in English were selected.

## Abbreviations

CCL: C-C motif chemokine ligand
DAMP: Damage-associated molecular pattern
ERK: Extracellular signal-regulated kinase
FABP4: Fatty acid binding protein
FFA: Free fatty acids
FNDC4: Fibronectin type III domain-containing protein 4
Glut: Glucose transporter
HMGB1: High mobility group box 1 protein
HOMA-IR: Homeostasis model assessment of insulin resistance
IFN: Interferon
IL: Interleukin
ILC: Innate lymphoid cell
iNKT: Innate NK T cell
IRE-1: Inositol-requiring protein 1
MAIT: Mucosa-associated invariant T cell
MCP-1: Macrophage chemotactic protein 1
MHC: Major histocompatibility complex
NF-kB: Nuclear factor “kappa-light-chain-enhancer” of activated B cells
NK: Natural killer
NLRP3: NOD-, LRR-, and pyrin domain-containing protein 3
PAI: Plasminogen activator inhibitor
PPARγ: Peroxisome proliferator-activated receptor gamma
SAM: Sympathetic neuron-associated macrophage
SASP: Senescence-associated secretory phenotype
Th: Helper T cell
TGF: Tumor growth factor
TLR: Toll-like receptor
TNF: Tumor necrosis factor
Treg: Regulatory T cell
UCP: Uncoupling protein
